# What is the Real Cost of Intraoperative Floppy Iris Syndrome in Cataract Surgery?

**DOI:** 10.18502/jovr.v18i1.12735

**Published:** 2023-02-21

**Authors:** Argyrios Tzamalis, Chrysanthos D. C hristou, Efthymia Prousali, Asimina Mataftsi, Nikolaos Ziakas

**Affiliations:** ^1^2nd Department of Ophthalmology, Aristotle University of Thessaloniki, Papageorgiou General Hospital, Thessaloniki, Greece

## Dear Editor,

Since its original description by Chang and Campbell in 2005, intraoperative floppy iris syndrome (IFIS) has been widely established as one of the most challenging conditions for cataract surgeons.^[1]^ Numerous studies have been published proving that the appearance of IFIS significantly increases the risk of intraoperative complications.^[2]^ However, there is no report so far in the literature dealing with the economic impact of IFIS in cataract surgery.

We conducted a retrospective analysis of the medical and financial records of all cases that underwent phacoemulsification surgery in a tertiary-care ophthalmology department during year 2019. Data regarding patient demographics, phacoemulsification metrics, surgical time, complications, and postoperative follow-ups were retrieved from the electronic patient records and the cost of all consumables charged in every case was recorded from the individual billing form that was automatically produced for every surgery. The surgical duration was recorded by an independent theatre nurse in each case. The timer was started upon the first incision and the endpoint was defined as the removal of the surgical drape. The study was performed according to the Tenets of the Declaration of Helsinki after approval of the Institutional Review Board.

**Table 1:** Average cost of surgical consumables and surgical duration in all cataract surgeries along with respective comparison between cases with and without IFIS.


	orange**IFIS** **(** ***n*** ** = 48)**	orange**Non-IFIS** **(** ***n*** ** = 1246)**	orange ***P*** -value ${}^{*}$
**Cost of surgical consumables ** **(Euros - €)**	420.2 ± 146.6	359.9 ± 87.5	< 0.0001
**Surgical time** **(min)**	30.9 ± 23.2	21.4 ± 14.8	< 0.0001
**Extra cost from surgical consumables per case** **(Euros - €)**	60.3	
**Extra cost from prolonged surgical time per case** **(Euros - €)**	41.04	
**Total extra cost per case** **(Euros - €)**	**101.34**	
	
	
white<bcol>4</ecol> ${}^{*}$ Assessed with Student's *t*-test. IFIS, intraoperative floppy iris syndrome

**Table 2:** Average cost of surgical consumables and surgical duration in uneventful cataract surgeries along with respective comparison between cases with or without IFIS.


	orange**IFIS** (*n* = 39)	orange**Non-IFIS** (*n* = 1199)	orange ***P*** **-value ${}^{ⱡ}$ **
**Cost of surgical consumables ** **(Euros - €)**	384.2 ± 48.3	352.8 ± 47.1	0.0003
**Surgical time** **(minutes)**	23.2 ± 11.6	16.7 ± 8.4	< 0.0001
**Extra cost from surgical consumables per case** **(Euros - €)**	31.4	
**Extra cost from prolonged surgical time per case** **(Euros - €)**	28.08	
**Total Extra cost per case** **(Euros - €)**	**59.48**	
	
	
white<bcol>4</ecol> ${}^{ⱡ}$ Assessed with Student's *t*-test. IFIS, intraoperative floppy iris syndrome

In total, 1294 cases of 1178 cataract patients (mean age = 73.8 ± 8.9 years), with (*n* = 48) or without (*n* = 1246) a recorded IFIS of any severity, were identified and enrolled in a multivariate analysis. As per our departmental policy, the presence of IFIS was defined and further classified as the intraoperative occurrence of any of the following three signs according to the grading system proposed by Chang and Campbell: progressive miosis; billowing of the iris; iris prolapse through incisions.^[3]^ Patients with one of these three clinical signs were designated as having IFIS. The average cost of surgical consumables charged among all cataract operations was 362.1 ± 90.9 €, while the mean duration of surgery was 21.75 ± 15.1 min ( ' ). No statistically significant differences in cost or surgical time were noted regarding age, gender, medical history, medication intake, or other ophthalmic conditions such as pseudoexfoliation, glaucoma, and cataract grading (*P* > 0.05). Cases that developed IFIS demonstrated a significantly higher cost of surgical consumables (CostIFIS = 420.2 ± 146.6 € vs CostNON-IFIS = 359.9 ± 87.5 €, *P* < 0.0001) as well as a longer duration of surgery (TimeIFIS = 30.9 ± 23.2 ' vs TimeNON-IFIS = 21.4 ± 14.8 ' , *P* < 0.0001) [Table 1]. This difference remained significant even when excluding all complicated cases (4.32% in total; 18.75% in IFIS cases) such as posterior capsular rupture, zonular dehiscence, nucleus drop, and iris trauma [Table 2]. Notably, no pupillary expansion devices were used in any of the cases included in the analysis. The increased cost in cases of IFIS was mainly due to the use of additional consumables such as extra OVDs, dyes, single-use instruments, as well as anterior vitrectomy in complicated cases.

Moreover, we attempted to assess the additional cost that the aforementioned longer duration of surgery had imposed in cases with IFIS. The cost of operating time in cataract surgery has already been evaluated in several cost-effectiveness analyses, ranging from 0.56 to 2.36 €/min (average = 1.21) in European countries^[3]^ and from 8.3 to 11.24 US dollars ($)/min in the United States.^[4,5]^ The cost per minute of surgical time was evaluated by the departmental accounting officers and found to be 4.32 €/min. It was calculated by dividing the total minutes of all surgeries into their non-supply cost (sum of salaries and wages of all hospital personnel included in cataract surgery) for the single fiscal year of 2019, based on previous reports.^[4,5]^ Subsequently, the appearance of IFIS imposed an extra cost of 28.08 € per patient as an average in non-complicated surgeries and 41.04 € per patient when all cases were considered. Summing up increased consumables and increased duration resulted in a total extra cost of 59.48 € in uneventful cases and 101.34 € when complicated cases were also included [Tables 1 & 2].

It is of note that the average surgical duration of phacoemulsification in this study was found to be somewhat longer than in other similar studies. This may be attributed to the fact that our analysis also included cases performed by trainees, who, as expected, required more time to complete the surgery. However, the occurrence of IFIS and the respective intraoperative complications rate did not yield any statistically significant difference between senior surgeons and trainees (*P *= 0.43 and *P *= 0.17, respectively).

Further limitations of this study include its retrospective nature that may have had an impact on our results. Although an electronic record is automatically produced for each surgery, it is possible that some cases of mild IFIS may have been ignored or misidentified and consequently, not recorded. Moreover, our study included cases performed by surgeons of various experience levels, and, thus, differences in phacoemulsification techniques and consumables used may have induced some bias. However, all surgeons that participated in the study initially utilized the same phaco technique (divide & conquer) and device (Centurion Vision System, Alcon).

In conclusion, the appearance of IFIS seems to have a substantial economic impact in cataract surgery as it increases the cost of surgical consumables and the time needed to complete the procedure, even in uneventful cases. Cataract surgeons should be aware of cases prone to develop IFIS and they are justified from a financial point of view to use all appropriate measures to prevent and manage floppy iris avoiding extra costs and devastating ocular complications.

##  Financial Support and Sponsorship

None.

##  Conflicts of Interest

None.
